# Inter-ethnic differences in efavirenz CNS toxicity – role of cytochrome P450 2B6 polymorphisms

**DOI:** 10.1186/1471-2334-12-S1-O17

**Published:** 2012-05-04

**Authors:** Lawrence S Lee, Gaik Hong Soon, Yik Ying Teo

**Affiliations:** 1 Department of Medicine, National University of Singapore, Singapore 119228, Singapore; 2 Department of Statistics & Applied Probability, National University of Singapore, Singapore 117546, Singapore

## Background

Highly active antiretroviral therapy regimens that include efavirenz are effective, but adverse events are common, especially central nervous system (CNS) toxicities. We previously showed increased discontinuation rates of efavirenz due to CNS toxicities in Malay and Chinese patients in our Treat Asia HIV Observational Database (TAHOD) database. We postulate that these differences may be due to genetic differences.

## Methods

We performed genome wide genotyping with the Illumina 1M and the Affymetrix 6.0 microarrays in healthy volunteers from the 3 major ethnic groups in Singapore, consisting of Chinese (mainly of southern Chinese origin), Malays and Indians (mainly of southern Indian origin). Genetic information was available from 95 Chinese, 89 Malays and 82 Indians.

## Results

Allele frequencies were obtained for the rs3745274 single nucleotide polymorphism coding for the cytochrome P450 2B6 (CYP2B6) 516 G>T mutation. The allele frequency of the mutant gene was found in 22.1% in Chinese, 37.6% in Malays and 37.8% in Indians. Homozygous TT mutations were found in 4.2% in Chinese, 12.4% in Malays and 12.2% in Indians. This homozygous TT mutation frequency is higher than that found in the European American HIV-infected population, which was reported to be 3%.

## Conclusion

Our study showed significant ethnic differences in CYP2B6 516 G>T mutations in Singapore. The frequencies of these mutations are significantly higher than in Caucasian populations. This finding may explain the higher discontinuation rate of efavirenz due to CNS side effects. Further studies with studying the association of CYP2B6 genotype with CNS toxicities and efavirenz concentrations are indicated.

